# Supporting Cell-Based Tendon Therapy: Effect of PDGF-BB and Ascorbic Acid on Rabbit Achilles Tenocytes In Vitro

**DOI:** 10.3390/ijms21020458

**Published:** 2020-01-10

**Authors:** Olivera Evrova, Damian Kellenberger, Maurizio Calcagni, Viola Vogel, Johanna Buschmann

**Affiliations:** 1 Division of Plastic Surgery and Hand Surgery, University Hospital Zurich, Sternwartstrasse 14, 8091 Zurich, Switzerland; olivera.evrova@gmail.com (O.E.); Maurizio.Calcagni@usz.ch (M.C.); 2 Laboratory of Applied Mechanobiology, ETH Zurich, Vladimir-Prelog-Weg 4, 8093 Zurich, Switzerland; Damian.Kellenberger@hest.ethz.ch (D.K.); Viola.Vogel@hest.ethz.ch (V.V.)

**Keywords:** tenocytes, PDGF-BB, ascorbic acid, proliferation, gene expression, collagen I, fibronectin

## Abstract

Cell-based tendon therapies with tenocytes as a cell source need effective tenocyte in vitro expansion before application for tendinopathies and tendon injuries. Supplementation of tenocyte culture with biomolecules that can boost proliferation and matrix synthesis is one viable option for supporting cell expansion. In this in vitro study, the impacts of ascorbic acid or PDGF-BB supplementation on rabbit Achilles tenocyte culture were studied. Namely, cell proliferation, changes in gene expression of several ECM and tendon markers (collagen I, collagen III, fibronectin, aggrecan, biglycan, decorin, ki67, tenascin-C, tenomodulin, Mohawk, α-SMA, MMP-2, MMP-9, TIMP1, and TIMP2) and ECM deposition (collagen I and fibronectin) were assessed. Ascorbic acid and PDGF-BB enhanced tenocyte proliferation, while ascorbic acid significantly accelerated the deposition of collagen I. Both biomolecules led to different changes in the gene expression profile of the cultured tenocytes, where upregulation of collagen I, Mohawk, decorin, MMP-2, and TIMP-2 was observed with ascorbic acid, while these markers were downregulated by PDGF-BB supplementation. Vice versa, there was an upregulation of fibronectin, biglycan and tenascin-C by PDGF-BB supplementation, while ascorbic acid led to a downregulation of these markers. However, both biomolecules are promising candidates for improving and accelerating the in vitro expansion of tenocytes, which is vital for various tendon tissue engineering approaches or cell-based tendon therapy.

## 1. Introduction

Different cell-based therapies, including biological and decellularized tissues [1,2], cell-seeded grafts from natural and synthetic biomaterials [3,4] or stem cell-based approaches [5] have been explored for the treatment of tendinopathies and tendon injuries. Among them, there are several reports on the use of mesenchymal stem cells (MSCs) for tendon cell-based therapy [6,7], or as a cell source for tissue engineered grafts and constructs where tenogenic differentiation was studied [8,9]. On the other hand, there are fewer reports exploring tenocytes for cell-based therapy [1,10,11], which might be due to the scarcity of donor tendons or loss of phenotype during in vitro expansion [12]. However, several studies describe the positive effects of autologous human tenocyte transplantation on tendon healing [13,14,15]. This suggests the need for tenocyte expansion and optimization of the expansion protocols before application, addressing increased proliferation, matrix synthesis, and phenotype retention in order to make them suitable for cell therapy. While the application of stem cells and cues for tenogenic differentiation have been reported extensively [16,17,18,19], the use of tenocytes to treat tendon diseases is currently not investigated widely. This may be due to two facts: first, tenocytes occur in relatively low numbers in tendon tissue and have to be expanded in vitro prior to application; second, tenocytes may undergo a phenotypic drift in culture, losing typical tendon markers during passaging, and consequently providing a limited range of low passages convenient for cellular therapy [20]. In comparison to stem cells, tenocytes are the main cell component of the tendon tissue. They are fully differentiated and functional within the tendon connective tissue [21], and can be easily extracted from parts of tendon tissue, although donor-site morbidity has to be taken into account. Nevertheless, tenocytes present an attractive cell source for tendon tissue engineering applications.

One way to improve cell-based tendon therapy is to perform a pre-treatment on the cell culture in order to increase cell proliferation and matrix synthesis before an application. Strategies for pre-treatment of tenocyte or MSCs cell culture include mechanical stimulation [8,22], three-dimensional spheroid cultures [23], macromolecular crowding [24] or biochemical supplementation of the culture medium [12,25]. From biochemical supplementation, stimulation by different growth factors, including transforming growth factor-beta (TGF-β) [8], bone morphogenetic protein-12 (BMP-12) [26], connective tissue growth factor (CTGF) [27], and platelet-derived growth factor-BB (PDGF-BB) [28] has been explored. Small bioactive molecules like ascorbic acid (AA), i.e., Vitamin C [29,30,31] or glutamine [12] have also been investigated. Interestingly, bioactive molecules are often investigated concerning their effect on stem cell tenogenic differentiation under serum+ conditions [18,32,33,34] and serum-free conditions [35,36], while effects on tenocytes have not been investigated in-depth so far, and only few reports for human or animal tenocytes exist [37,38,39]. Additionally, it is beneficial to determine the effects of biomolecules on tenocyte culture not only for pre-treatment for cell-based therapy but also since tenocytes are the main cell population affected by the delivery of drugs explored for aiding tendon tissue healing and repair.

Taking this into account, we investigated the effects of two biomolecules on rabbit Achilles tenocyte culture in vitro; namely PDGF-BB and AA. PDGF-BB is well known for its mitogenic, chemotactic and angiogenic properties and has been explored as a growth factor to help the tendon healing process upon injury or rupture [28]. In addition, PDGF-BB also has an important role in stimulating the production of other growth factors, such as insulin-like growth factor (IGF-1), that in turn can also support tendon healing [40]. In contrast, TGF-β may lead to aberrant scar formation [41,42,43], while BMPs are reported to turn stem cell towards tenogenic lineage, but less to boost matrix formation. However, the PDGF-BB effects on tenocyte matrix synthesis or gene expression over time have not been investigated in detail so far.

On the other hand, AA is known to promote collagen biosynthesis [44,45], and it has also been suggested as a molecule that could help tendons to heal [46]. While its effects on matrix synthesis are known to some extent [30], what changes its supplementation might bring to the phenotype of tenocytes is less known.

Hence, the hypotheses in this study were:

- PDGF-BB and AA increase the proliferation of tenocytes.
- PDGF-BB and AA enhance extracellular matrix synthesis.
- PDGF-BB and AA change the gene expression of tendon markers.
- PDGF-BB and AA released from bioactive scaffolds support tenocyte growth and ECM synthesis.

## 2. Results

### 2.1. AA and PDGF-BB Supplementation Increase Tenocyte Proliferation

**Figure 1:**
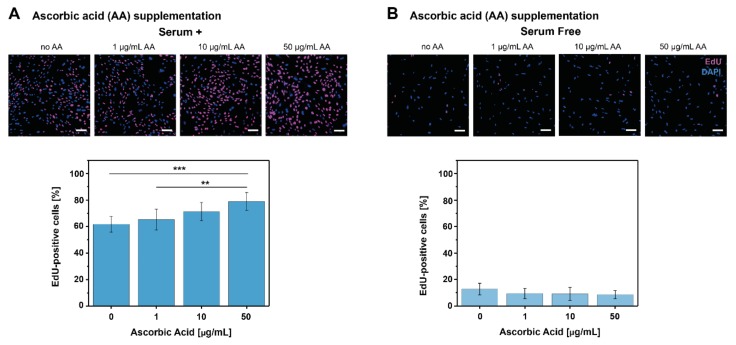
Effect of AA on tenocyte proliferation. Representative CLSM images from the EdU proliferation assay and EdU-positive cells [%] for each AA concentration (μg/mL) tested in (**A**) serum+ conditions and (**B**) serum-free conditions. The effect of different PDGF-BB concentrations on tenocyte proliferation assessed by the EdU assay is presented in Figure S1. The total EdU-positive cells [%] was calculated as EdU positive cells (pink) in relation to the total cell number (DAPI stained, blue). Scale bars: 100 μm. (Data is shown as mean ± standard deviation, ** *p* < 0.01, *** *p* < 0.001 obtained by one-way ANOVA).

In order to assess the effect of both biomolecules on cell culture proliferation, tenocytes cultured in both serum+ and serum-free medium were supplemented with different concentrations of PDGF-BB and AA. Cell proliferation was evaluated by the EdU assay, and data for AA treatment is shown in Figure 1. Data for the PDGF-BB treatment is available from a previous study [47] and is shown in Figure S1. This data obtained with the EdU assay allowed observation of the short term effect (24 h post stimulation) of both molecules on tenocyte proliferation. Under serum+ conditions, AA concentrations (1–50 μg/mL) led to a dose-dependent increase in tenocyte proliferation (% of EdU-positive cells). However, only 50 μg/mL AA led to a significantly higher cell proliferation when compared to no supplementation or 1 μg/mL AA (Figure 1A). Under serum-free conditions, the tenocyte proliferation was not affected by any AA concentration, with very low proliferation rates of approximately 10% (Figure 1B). On the other hand, there was a significant, dose-dependent increase in cell proliferation with 10–50 ng/mL PDGF-BB concentrations under serum-free conditions (Figure S1B), while no significant differences were observed under serum+ conditions for any PDGF-BB concentration (Figure S1A).

### 2.2. Tenocyte Morphology and ki-67 Expression Changes under Different Conditions over Time

**Figure 2:**
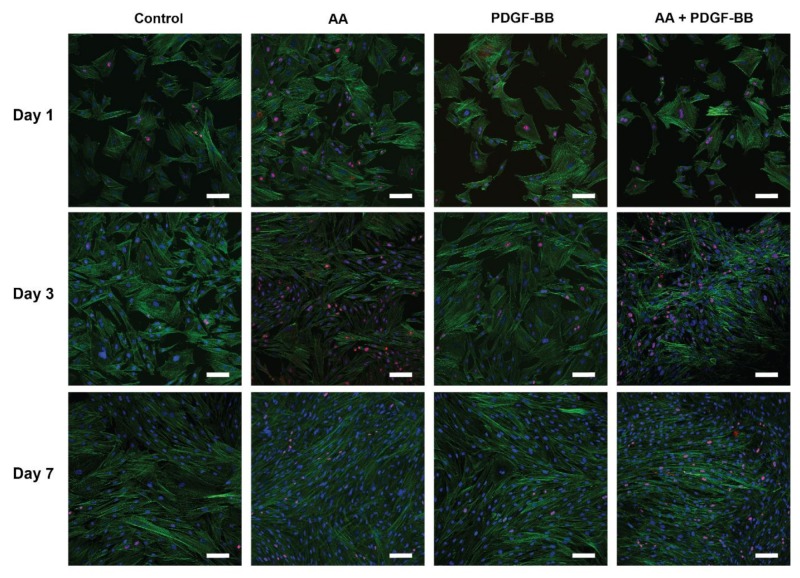
Effect of AA and PDGF-BB on tenocyte proliferation and morphology in cell culture. Representative CLSM images of tenocytes cultured in serum+ medium for a period of 7 days and supplemented with either AA (10 μg/mL), PDGF-BB (25 ng/mL), combination of AA (10 μg/mL) + PDGF-BB (25 ng/mL), or untreated (control). Cells were stained with phalloidin (green) for visualizing the cytoskeleton, ki-67 (red) as a proliferation marker, and DAPI (blue). Scale bars: 100 μm.

Phalloidin staining for cell morphology visualization through actin cytoskeleton staining, together with ki-67 staining as a proliferation marker, was performed for tenocytes grown in medium supplemented with AA (10 µg/mL), PDGF-BB (25 ng/mL) or both molecules (10 µg/mL AA + 25 ng/mL PDGF-BB), for 7 days (day 1, 3 and 7) (Figure 2). In Figure 2, changes in cell morphology over time in serum+ conditions can be observed. While on day 1 the cell culture for all the conditions was a mixture of spread out cells and more elongated ones, the cell morphology changed in the supplemented cultures to a more longish, spindle-shaped type on day 3, which was even more pronounced on day 7. Compared with the control (no supplementation) and PDGF-BB treated cell cultures, the addition of AA revealed a higher cell confluence over time, with extremely thin and long cells on day 7. Similarly, based on cell density, the same effect was observed for the culture with AA + PDGF-BB treatment. In contrast, cells in the control condition on day 7 had changed to a longish morphology when compared to earlier time points, but still were not as elongated and dense as in the presence of AA or PDGF-BB (qualitative inspection).

From the representative images in Figure 2, it can be observed that AA, PDGF-BB, and combined supplementation led to an increasing number of ki-67 positive cells over time, with the highest cell proliferation on day 3, when compared to the control condition.

### 2.3. AA and PDGF-BB Supplementation Differently Affected Expression of Tendon Specific Markers and ECM Proteins

To determine what effect AA and PDGF-BB supplementation might have on gene expression in tenocytes, the transcription of different tendon-specific markers, ECM proteins, and genes involved in ECM turnover was analyzed.

**Figure 3:**
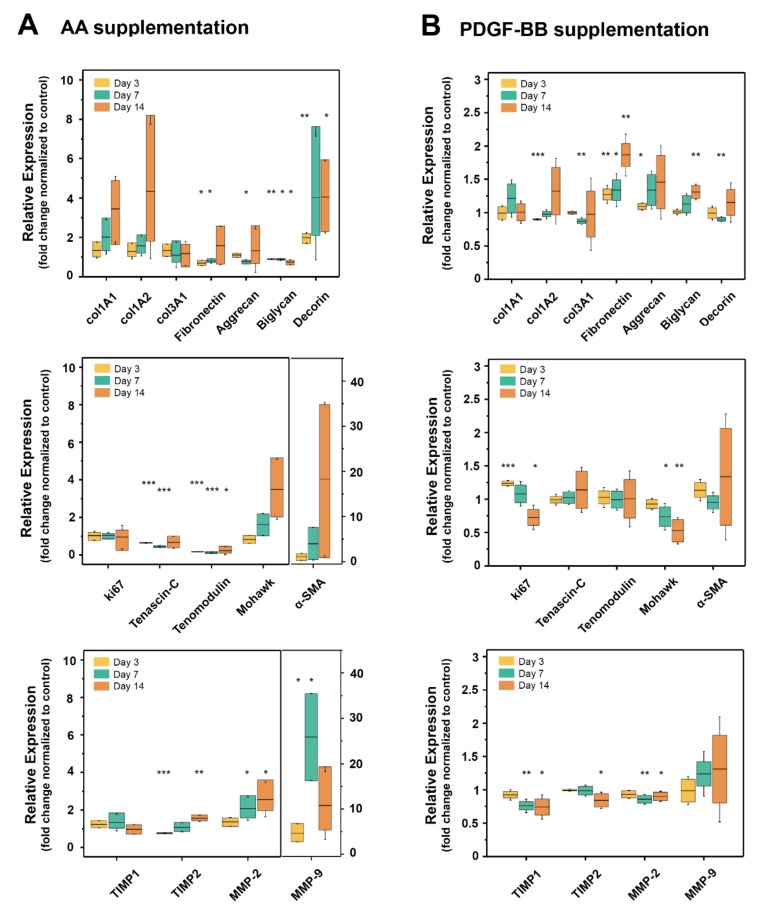
Gene expression of tenocytes supplemented with AA or PDGF-BB over 14 days in 2D cell culture. (**A**) Box-plots of time-dependent (day 3, 7 and 14) relative gene expression in tenocytes supplemented with AA (50 μg/mL), normalized to non-stimulated tenocytes and (**B**) box-plots of time-dependent (day 3, 7 and 14) relative gene expression in tenocytes supplemented with PDGF-BB (25 ng/mL), normalized to non-stimulated tenocytes. In the experiments with AA, tenocytes isolated from three different rabbits were used (biological replicates *n* = 3), while for PDGF-BB experiments, cells from four different rabbits were used (biological replicates *n* = 4). Technical replicates in cell culture were performed in triplicate and in PCR experiments in duplicate. Significant differences were based on unpaired *t*-tests for each time point (whiskers on the box-plots represent standard deviation, * *p* < 0.05, ** *p* < 0.01, *** *p* < 0.001).

During tendon repair, there is usually a 6-week cellular phase after an initial inflammatory phase. In the cellular phase, where tenoblasts invade the wound site, among other cells, upregulation of tenomodulin is highly important because tenomodulin regulates tenocyte proliferation and the growth of collagen fibers [48,49,50]. In addition, the transiently expressed tenascin-C plays a pivotal role in this phase, as it modulates proliferation, fiber alignment, and orientation [51]. Although Mohawk is particularly involved in the tenogenic differentiation of stem cells [52] by preventing osteogenic and chondrogenic differentiation [53], it also supports tendon repair by improved collagen deposition [54]. As for the remodeling of the matrix and the wound repair, after the initial fibronectin deposited acts as a template, collagen III and later on collagen I are deposited by the tendon cells. Proteins, such as biglycan and decorin, play an important role as they bind water and are therefore add to the elasticity of the tendon matrix [55]. For remodeling, matrix metalloproteases (MMPs) as well as their inhibitors (TIMPs) are of high importance [56] and are therefore included in the following gene expression analysis. The expression of the genes of interest was analyzed at three different time points, i.e., day 3, 7 and 14, and the relative expression was normalized to the control (non-supplemented serum+ medium) (Figure 3).

Upon AA treatment, a trend of increase in the expression of collagen I (col1A1 and col1A2), Mohawk and alpha-SMA over time can be observed (Figure 3A, first and second panel), but with big variation among results gained from tenocytes of different rabbits. Fibronectin was significantly downregulated in the first week of AA treatment, while on day 14 there was an increase in its expression, but with large variation among animals. Both aggrecan and biglycan were downregulated in the first week, but only biglycan downregulation was significant also on day 14. Tenascin-C and tenomodulin expression were significantly downregulated, while decorin was significantly upregulated. Collagen III expression (col3A1) showed a slight decrease over time but without significant changes, similar to ki67 expression. MMP-2 expression was upregulated significantly on day 7 and 14, which was accompanied by a very similar and significant increase in TIMP-2 gene expression on day 3 and 14. While TIMP-1 expression was not affected by AA supplementation, MMP-9 expression was significantly increased on day 7, followed by a decrease in expression on day 14.

On the other hand, PDGF-BB supplementation did not lead to significant changes in the collagen I (col1A1 and col1A2) expression over time (Figure 3B, first panel). Although there was a significant downregulation in col1A2 on day 3 followed by a trend of upregulation on day 7 and 14, there was no significant change in col1A1 for all time points considered here. Collagen III was significantly downregulated on day 7, but without significant change on day 14. In contrast, fibronectin was significantly upregulated over time, starting on day 3 and was even more pronounced on day 14 (two-fold increase). Aggrecan was initially upregulated (day 3), without significant changes at later time points, while biglycan expression showed a significant increase on day 14. As for decorin, another typical tendon marker besides biglycan, there was a small but significant downregulation on day 7, without significant changes at later time points. 

The proliferation marker ki67 was significantly upregulated on day 3, suggesting increased proliferation at this time point. However, expression decreased over time with significant downregulation on day 14 (Figure 3B, second panel). The most substantial effect of PDGF-BB supplementation on the tendon-specific markers (tenomodulin, Mohawk, and tenascin-C) was observed on the Mohawk gene expression, where significant downregulation was already observed on day 7, and the trend continued on day 14. Expressions of tenomodulin and tenascin-C were not significantly affected by PDGF-BB supplementation. MMP-9 expression varied a lot throughout all time points, but it showed a slight upregulation trend, while MMP-2 expression showed a significant downregulation on days 7 and 14. A downregulation trend was also observed for TIMP1 and TIMP2 with significant changes at later time points, i.e., days 7 and 14.

### 2.4. AA Supplementation Increased ECM Deposition by Tenocytes

**Figure 4:**
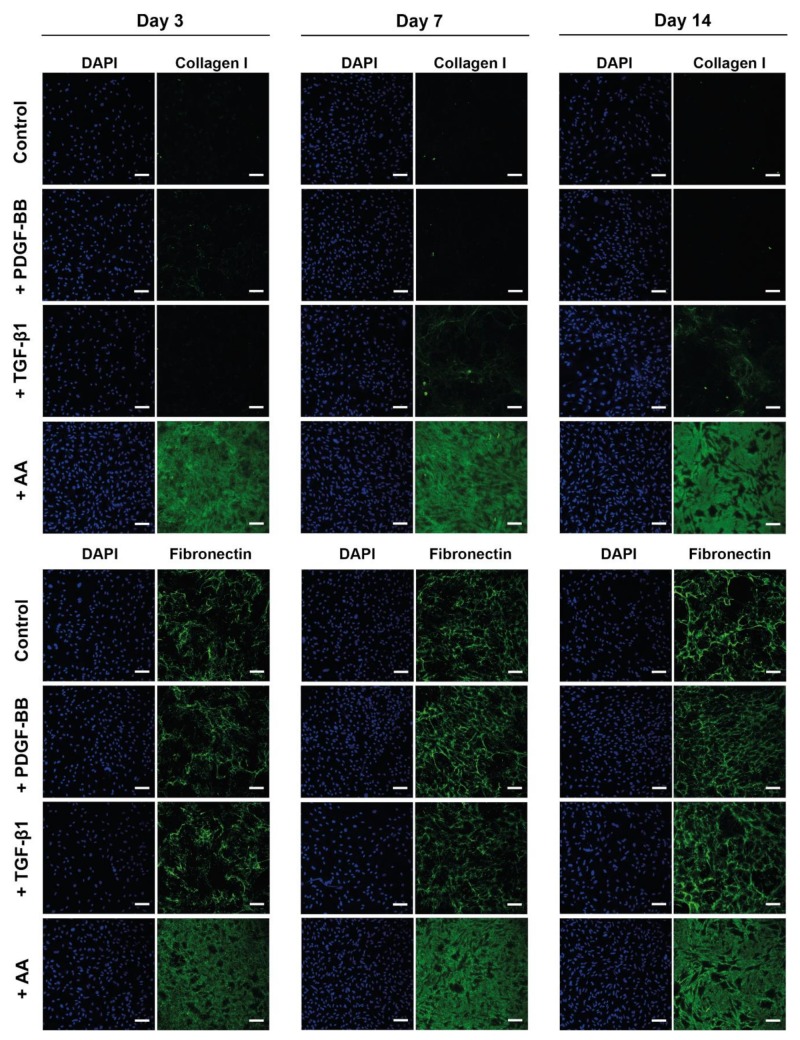
Extracellular matrix deposition by tenocytes stimulated with PDGF-BB, AA or TGF-β1. Representative CLSM images of collagen I or fibronectin staining (green) and cell nuclei DAPI staining (blue) of rabbit tenocytes cultured in vitro in 2D, treated with PDGF-BB (50 ng/mL), TGF-β1 (10 ng/mL), AA (50 μg/mL) or untreated (control) on day 3, 7 and 14. For additional data of the same experiment performed with tenocytes isolated from two other animals and for quantification of the fibronectin deposition under the different treatments, we refer to the figures in the supporting information. Scale bars: 100 μm.

Figure 4 shows the effect of AA and PDGF-BB on the ECM synthesis in tenocyte cell culture, i.e., collagen I and fibronectin deposition by tenocytes over time (day 3, 7 and 14). As a comparison, TGF-β1 was also used as a treatment because it is known to stimulate ECM synthesis [57,58]. All the conditions were compared to tenocytes grown only in serum+ culture medium (control), and tenocytes isolated from three different rabbits were assessed (Figure 4, Figures S2 and S3). Since the medium in all conditions was supplemented with 30 μg/mL fibronectin isolated from human plasma [59]; thus, the staining of fibronectin detects both newly synthesized fibronectin by the rabbit tenocytes, as well as the assembled fibronectin supplemented in the medium. In all tenocyte cultures, the fibronectin matrix is denser in the PDGF-BB, TGF-β1, or AA conditions when compared to the control, and the density of the fibronectin matrix increases over time, from day 3 to 14. Moreover, in the TGF-β1 condition, on average, the cell number is lower than in the PDGF-BB or AA condition (due to the proliferative effect of both molecules). However, the fibronectin matrix is as dense as in the other two conditions, suggesting that TGF-β1 supplementation leads to an increase in fibronectin deposition by tenocytes. Quantitative analysis of the fibronectin signal normalized to the total cell number in each condition (Figure S4) indeed showed an increase in fibronectin deposition upon TGF-β1 stimulation when compared to the other conditions, especially after 14 days in culture. These results were observed for tenocytes isolated from all three animals. The increase in fibronectin deposition was also confirmed for the AA treatment. On the other hand, normalization of the fibronectin signal to the total cell number revealed that while a denser matrix was present in the PDGF-BB condition, the fibronectin deposited per cell did not differ a lot from the control condition and it varied between the different tenocyte isolations (Figure S4).

Upon PDGF-BB supplementation, an increase in cell number (DAPI staining) was observed when compared to the control condition, but no collagen I synthesis was detected in any of the samples at all time points, for any of the cell cultures from all animals. TGF-β1 treatment led to an increase in collagen I on day 7 and day 14, but only for tenocytes isolated from two animals (Figure 4 and Figure S2). As early as day 3, no visible collagen I deposition was present in any of the samples with TGF-β1 treatment. On the other hand, AA addition led to significant collagen I deposition already on day 3. In the presence of AA, tenocytes from all animal donors deposited collagen on day 3, 7 and 14, and over time the collagen I matrix is becoming denser as observed in Figure 4, Figures S2 and S3.

In addition to assessing AA and PDGF-BB supplementation in a cell medium and 2D environment, it was also investigated what the effect would be of these molecules when they are incorporated and released over time from electrospun scaffolds, in a 3D environment. Biocompatible scaffolds can play a role as a key delivery platform for cell therapy in various tissue engineering approaches, and bioactive scaffolds would also be beneficial for this purpose. A convenient and easy method for production of bioactive scaffolds is emulsion electrospinning, which allows a sustained release profile of the incorporated molecule over time [28]. Previous research has shown that both AA and PDGF-BB can be successfully incorporated into electrospun scaffolds and released in a sustained manner over a period of 30 days [47].

**Figure 5:**
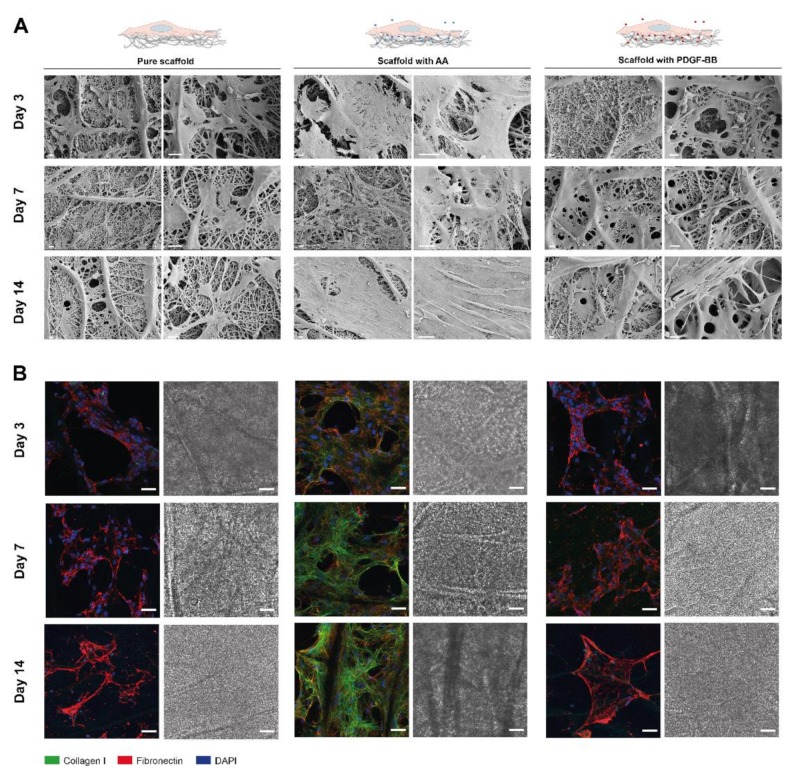
Tenocytes cultured on pure and bioactive scaffolds over a period of 14 days. (**A**) SEM images of tenocytes, seeded on bioactive electrospun DP scaffolds, containing either AA, PDGF-BB (bioactive scaffolds) or none of them (pure scaffold). The cell morphology and ECM deposition overtime is observed on the SEM images. (**B**) Representative CLSM images of collagen I (green), fibronectin staining (red) and cell nuclei DAPI staining (blue) of rabbit tenocytes cultured onto the electrospun scaffolds. Scale bars: (**A**) 20 µm and (**B**) 50 μm.

Primarily, cell culture over time and ECM production onto the scaffolds were examined by SEM, as well as collagen I and fibronectin staining (Figure 5). Representative SEM images in Figure 5A show a significant increase in ECM production on the scaffolds releasing AA, which is already visible on day 3. Continuous coverage of the scaffold surface by cells and deposited ECM is visible on day 14, which is not the case in the control group (pure scaffolds) or on scaffolds with incorporated PDGF-BB. The staining of collagen I and fibronectin confirms these results (Figure 5B). While no collagen I deposition is observed for tenocytes seeded onto pure scaffolds and scaffolds with PDGF-BB, a significant deposition is visible when cells were cultured on the bioactive scaffolds containing AA. Collagen I deposition stimulated by the released AA over time from the scaffolds is visible already on day 3. These results suggest that AA is released over time from the bioactive electrospun scaffolds and that it retains its bioactivity by stimulating cell proliferation and ECM deposition.

## 3. Discussion

In order to treat tendon diseases and support tendon regeneration, cell-based therapy, as well as other tissue engineering approaches, are viable options. Although it is self-evident that tenocytes might be a valuable cell source for tissue engineering approaches to treat tendon injuries and diseases, a pre-treatment step in culture could enhance their regenerative potential. Particular attention during the selection of pre-treatment options is paid to accelerate cell expansion by increasing cell proliferation. Therefore, the main aim of this study was to assess the effects of PDGF-BB and AA in vitro on rabbit Achilles tenocyte culture, as they have been proposed as biomolecules of interest for tendon healing, as well as biochemical supplementation, which affects cell proliferation and ECM synthesis of tenocyte culture.

PDGF-BB has been studied as a growth factor of interest to be delivered to support tendon healing upon injury/rupture due to its mitogenic, chemotactic and angiogenic properties [28]. There are studies on tenocyte proliferation, dealing with the effects of PDGF-BB, but also of other growth factors [60], with a particular emphasis on using tenocytes for the population of scaffold materials or for re-populating previously decellularized matrices [61]. On the other hand, the efficacy of AA supplementation after musculoskeletal injuries with respect to collagen synthesis has been investigated widely [62]. As a cofactor for prolyl hydroxylase and lysyl hydroxylase, AA plays a pivotal role in the synthesis of collagen [63,64] and, as such, can play an important role in the healing of connective tissue. Recently, a proof of concept study showed that AA led to better healing in a rat Achilles tendon injury model, particularly in combination with thyroid hormone T3 [65]; with a significant increase in cell proliferation, more regular fiber structure and higher collagen I to collagen III ratio, indicating an accelerated healing process [21]. Moreover, AA supplementation also stimulated Achilles tendon healing in rats after complete rupture [46]. Thus, in this study, the impact of both biomolecules on tenocyte proliferation, morphology, gene expression, and ECM deposition of rabbit Achilles tenocytes was assessed.

One of the findings in our study was a dose-dependent increase of tenocyte proliferation upon stimulation with both biomolecules, but under different medium conditions. When short term (24 h post supplementation) tenocyte proliferation was assessed, for PDGF-BB supplementation the effect was stronger under serum-free conditions, while for AA supplementation under serum+ conditions (Figure 1 and Figure S1). This could be due to the fact that the effect of PDGF-BB is masked by the presence of serum in the medium, which contains other growth factors and molecules that support cell growth, so its effect on tenocyte proliferation and DNA synthesis is clearly isolated under serum-free conditions. On the contrary, AA stimulation led to a visible, dose-dependent increase in cell proliferation only in serum+ medium. It has been shown in adipose-derived stem cells [66] or hepatocytes [67] that AA inserts its effect on proliferation by activating MAPK signaling, and it could be that in the short term (24 h) assessment of tenocyte proliferation, the effect is not strong enough.

Complimentary to the short term cell proliferation, tenocyte proliferation over time in serum+ medium was also assessed by looking at ki-67 expression as a proliferation marker. Cell proliferation in both treatments peaked on day 3 of incubation (Figure 2), and qualitatively PDGF-BB supplementation showed an increase in cell proliferation in serum+ condition as well.

These findings on the dose-dependent effect of both molecules on cell proliferation are in agreement with previous studies that have reported similar results. Similar concentrations of PDGF-BB (10–50 ng/mL) have been shown to promote tenocyte proliferation in vitro best [28,39,68,69] and also in combination with other growth factors, like basic fibroblast growth factor (bFGF) and insulin-like growth factor (IGF-1) [39,61]. In addition, other growth factors, such as VEGF, have also been tested. For that purpose, human tenocytes were cultivated in the presence of 5, 10 and 20 ng/mL VEGF, respectively [37]. Not only was proliferation significantly increased at 20 ng/ mL VEGF compared with the control, but also gene expression was significantly affected. A similar concentration of AA (10–50 μg/mL) led to an increase in tenocyte proliferation in previous studies as well [38]. Both molecules and a combination of them led to higher cell density over time (within a period of 7 days) and cell morphology change from more spread out to spindle-shaped cells (Figure 2), typical for tenocyte morphology, similar as reported in other studies as well [31].

An important finding in this study was the fact that both molecules affected the gene expression of several ECM proteins and tendon markers, but with different trends. AA led to an increasing trend in collagen I (col1A1 and cola1A2), Mohawk and α-SMA gene expression, and a significant increase in decorin, MMP-2, and TIMP2 expression (Figure 3A). Additionally, it led to a significant downregulation of fibronectin, aggrecan, biglycan, tenascin-C, and tenomodulin, the latter being two important tenocyte markers besides scleraxis [70] for which unfortunately a primer set for the rabbit species was unavailable. In contrast, tenocytes cultivated in the presence of PDGF-BB exhibited a prominent upregulation of fibronectin and biglycan after 2 weeks, while a significant downregulation of Mohawk, MMP-2, TIMP-2, and TIMP-2 was determined (Figure 3). As for the proliferation marker ki67, an upregulation was found on day 3, which was downregulated later on day 14, but this peak was timely coherent with the immunostaining performed for ki-67 in tenocyte culture (Figure 2). In terms of ECM deposition, as assessed by collagen I and fibronectin immunostaining in tenocyte culture over time, it was determined that AA strongly supported collagen I synthesis and also led to increasing fibronectin deposition, while PDGF-BB only led to a denser fibronectin matrix when compared to the control condition, but not to a visible collagen I deposition (Figure 4).

A similar trend was observed when tenocytes were seeded onto bioactive scaffolds that released in a sustained manner the biomolecules, where more prominent matrix formation and an increase in cell density were visible on scaffolds with AA when compared to ones with PDGF-BB or pure scaffolds (control) (Figure 5). Such results stand in agreement with the findings from Piran et al., where PDGF-BB released from chitosan nanoparticles showed a significant increase in the proliferation of fibroblasts [71].

The increase of collagen I synthesis by AA supplementation observed at the protein level, was supported by the gene expression data as well, showing an upregulation of collagen I, standing in agreement with previous studies [31,62]. A similar upregulation trend was observed for Mohawk, which was suggested to play an important role in collagen I production in tenocytes [52,53,72]. Fibronectin gene expression was downregulated by AA supplementation, while qualitatively a slight increase was visible in fibronectin deposition in culture. In contrast, stimulation by PDGF-BB led to significant upregulation of fibronectin accompanied by a small increase in collagen I on day 14 and a significant downregulation of Mohawk expression, in agreement with a slight increase in fibronectin deposition in culture and no visible deposition of collagen I. These results suggest that PDGF-BB supplementation led to expression and deposition of fibronectin as a first matrix scaffolding component for further ECM components, and while this process was ongoing, no upregulation or increase in collagen I expression took place at subsequent time points. On the other hand, when collagen I synthesis was stimulated by AA, fibronectin expression was decreased. Since it is known that these two ECM components are in close interplay, these observations are in alignment with previous research on the dynamics of fibronectin and collagen I [73,74,75].

Another component that has interactions with fibronectin is the glycoprotein tenascin-C, which is transiently elevated during injury and regulated by mechanical stress [51,76]. Tenascin-C gene expression was downregulated by AA, while being upregulated by PDGF-BB supplementation (Figure 3). Since tenascin-C and fibronectin assembly are interdependent [77], simultaneous upregulation or downregulation of tenascin-C and fibronectin, as observed in each supplementation, was expected and confirmed. Another explanation for the opposite impact on the expression of this gene might be related to the activation of different signaling pathways. While PDGF-BB regulates tenascin-C expression via the PI3K-Akt signaling pathway [76], a major player for tenascin-C regulation is the homeobox protein family [78], and AA is known to interact with Nanog [79,80], a homeobox protein, via the JAK/STAT signaling pathway [79]. Hence, although not elucidated in detail up to date, AA may influence tenascin-C gene expression by other regulatory pathways than PDGF-BB does; and JAK/STAT pathway may play a role. In addition, AA has been reported to act via Wnt signaling [81,82], but the regulation of tenascin-C expression by the Wnt/β-catenin signaling pathway has not been reported for tenocytes yet, but only for macrophages [83] or smooth muscle cells [84].

A study by Orfei et al. on the in vitro induction of tendon-specific markers revealed induction of decorin protein expression by AA [30], which was observed in this study as well; AA stimulation led to a significant increase of decorin gene expression on day 7 and 14. However, PDGF-BB stimulation led to the opposite, at least on day 7, when a downregulation in decorin gene expression was observed. An opposite effect was observed for the gene expression of biglycan: downregulated by AA, but upregulated in the presence of PDGF-BB. Hence, biglycan and decorin experienced opposite changes in gene expression by the two biomolecules, and their changes may be dependent on the upregulation/downregulation and the dynamic interplay with the other matrix components.

Additionally, AA stimulation led to a significant increase in MMP-2 and TIMP-2 gene expression. Increases in MMPs are particularly associated with mechanical loading. For example, a repetitive stretching of human tenocytes for 5 days evoked a significant increase of MMP-2 on gene and protein expression levels [85,86]. Here, an increase under static cultivation conditions in vitro was found. MMP-2, the most abundant MMP [87], is known to degrade collagen fibers and its overexpression in aggressive tumors has been well described [88,89]. Its activation and activity are critically influenced by the tissue inhibitor of matrix metalloprotease, TIMP-2 [90]. As we found both genes significantly upregulated in the presence of AA, it can be concluded that matrix turnover and remodeling are enhanced in tenocyte culture supplemented with AA, which is of great importance during wound healing. These results also align with the observations in increased ECM synthesis, i.e., collagen I in the presence of AA.

Although several effects of PDGF-BB and AA on tenocyte culture in vitro could be shown in this study, there are some limitations. As mentioned above, scleraxis as a typical tenocyte marker gene could not be assessed because the gene for the rabbit species is not available. In addition, it would have been interesting to assess quantitatively the cell proliferation over a longer period, where we have assessed it only for 24 h with the EdU assay. It was only qualitatively assessed with ki-67 as a marker for proliferation over 7 days. Finally, different concentrations used in different experiments, such as for the extracellular matrix formation and gene expression, may hamper a direct comparison of the results.

In conclusion, this in vitro study shows that both biomolecules tested on rabbit Achilles tenocyte culture may act beneficially during tenocyte expansion prior to utilization for different tissue engineering approaches or application in vivo. AA and PDGF-BB enhanced tenocyte proliferation and accelerated the deposition of ECM components, such as collagen I and fibronectin, but with different dynamics in the ECM synthesis. AA led to a particularly strong increase in collagen I deposition. Interestingly, on the gene expression level, the two biomolecules led to different changes in some of the investigated markers and most notably affect collagen and fibronectin expression in opposite ways. While AA upregulated collagen I, Mohawk, decorin, MMP-2, and TIMP-2 expression, these markers were downregulated by PDGF-BB. On the other hand, PDGF-BB led to an upregulation of fibronectin, biglycan and tenascin-C, whereas AA showed downregulation of these markers. The interplay between the different ECM components and different signaling pathways involved may explain such outcomes. In order to conclusively determine the mechanisms standing behind these different findings, further studies will be needed. However, this knowledge can be used for improved tenocyte culture expansion and maybe a combination or subsequent supplementation of both biomolecules may lead to optimal tenocyte culture conditions. Like this, these findings may pave the way for improved cell-based tendon therapies.

## 4. Materials and Methods

### 4.1. Isolation of Rabbit Achilles Tenocytes

Rabbit tenocytes were isolated from Achilles tendons of New Zealand White rabbits using the cell migration method. Briefly, tendons were extracted from the animals and washed with Hank’s Balanced Salt Solution (1x HBSS with Ca^2+^ and Mg^2+^, Thermo Fisher Scientific, Rockford, IL, USA) with 200 µg/mL gentamicin (Biowest, Nuaillé, France) and 2.5 µg/mL amphotericin B (Biowest, Nuaillé, France). The tendons were cleaned from the surrounding tissue and the central part of the tendons was cut into very small pieces (< 2 mm) and washed three times in 1x HBSS buffer. Afterwards, multiple tissue pieces were placed into each tissue culture plate (Primaria^TM^, Corning, New York, NY, USA) and a drop of cell culture medium (Ham’s F12 (Biowest, Nuaillé, France), 10% FBS (Biowest, Nuaillé, France), 200 µg/mL gentamicin, and 2.5 µg/mL amphotericin B) was added onto each tissue piece. Tissues were allowed to attach onto the cell culture plates for 2 h at 37 °C and 5% CO_2_ before adding 10 mL of cell culture media in each plate. The plates with the tissues were not moved for the first 5 days, to decrease tissue detachment upon plate movement and to allow cells to start migrating out from the tissues. The first medium change was done after 5 days, and subsequently, the culture medium was changed every third day. After approximately 2 weeks, tissue pieces were removed from the plates, and cells were allowed to proliferate for 1 week more before cryopreservation. Cryopreserved rabbit tenocytes were thawed, resuspended in culture medium (Ham’s F12 with 10% FBS and 50 µg/mL gentamicin) and cultured at 37 °C and 5% CO_2_ with media being changed every second day. Tenocytes between passages 2 and 4 (P2–4) were used for all experiments (Approval by the veterinary office of Canton Zurich, ZH255/15, 21st April 2016.

### 4.2. EdU Proliferation Assay

The effect of AA (L-Ascorbic acid 2-phosphate sesquimagnesium salt hydrate, Sigma-Aldrich, Buchs, Switzerland) and recombinant human PDGF-BB (Peprotech, London, UK) on rabbit tenocytes were tested by an increase in cell proliferation assessed by the Click-iT EdU proliferation kit (Invitrogen, Carlsbad, CA, USA). The proliferative effect was studied in serum+ (Ham’s F12 with 10% FBS and 1% P/S) and serum-free (Ham’s F12, 1x RPMI vitamins solution (Sigma-Aldrich, Buchs, Switzerland), 1x non-essential amino acids solution (Invitrogen, Carlsbad, CA, USA) and 1% P/S) culture medium. AA concentrations of 1, 10 and 50 μg/mL and PDGF-BB (Peprotech, London, UK) concentrations of 10, 25 and 50 ng/mL were tested. Cells were seeded in 8-well μ slides, ibiTreat (Ibidi, Munich, Germany, growth area per well: 1.0 cm^2^) at 4 × 10^4^ cells mL-1 (300 μL per well). In the case of tenocytes supplemented with PDGF-BB, cells were first serum-starved for synchronization with a daily change of serum-free medium over 3 days. After 3 days, different PDGF–BB concentrations in serum-free or serum+ medium, respectively, together with 10 µM 5-ethynyl-2′-deoxyuridine (EdU) were added and incubated for 24 h at 37 °C and 5% CO_2_. In the case of tenocytes supplemented with AA under serum+ conditions, there was no cell synchronization performed with cell starvation, but rather cells were directly seeded in serum+ medium and after 24 h, different AA concentrations in serum+ medium together with 10 µM 5-ethynyl-2′-deoxyuridine (EdU) were added and incubated for 24 h at 37 °C and 5% CO_2_. Tenocytes supplemented with AA under serum-free conditions underwent cell synchronization with serum-starvation as the cells treated with PDGF-BB under serum-free conditions. After 3 days, different PDGF–BB concentrations in serum-free or serum+ medium, respectively, together with 10 µM 5-ethynyl-2′-deoxyuridine (EdU) were added and incubated for 24 h at 37 °C and 5% CO_2_. Cells were then fixed in 4% PFA, permeabilized with 0.1% TritonX-100 in 1x PBS and EdU stained according to the kit’s protocol. Cell nuclei were stained with 5 μg/mL-1 4′6-diamidino-2-phenylindole dilactate (DAPI) for 10 min. Samples were imaged with a confocal microscope (SP5, Leica Microsystems, Wetzlar, Germany) with 20x/0.7 N.A. objective and 30 random images were taken per sample (*n* = 6). The total EdU-positive cells [%] was calculated as EdU positive cells in relation to the total cell number in each image. Results are expressed as EdU-positive cells [%] ± standard deviations.

### 4.3. Tenocyte Morphology under Different Conditions

To assess the effect of AA and PDGF-BB on the morphology of tenocytes over time, as well as to additionally assess the cell proliferation at each time point, cells were cultured and stained with Phalloidin and ki-67. Cells were seeded in 8-well μ slides, ibiTreat (growth area per well: 1.0 cm^2^), in serum+ medium (1.4 × 10^4^ cells/mL (300 μL per well)). Cells in serum+ medium were cultured in the respective conditions (10 μg/mL AA, 25 ng/mL PDGF-BB, 10 μg/mL AA + 25 ng/mL PDGF-BB or just serum+ medium) over a period of 7 days and the medium was changed every 2 days. Desired PDGF-BB and AA concentrations were added freshly to the medium every time during medium exchange, for the whole duration of the experiment. Samples were fixed on day 1, 3 and 7 with 4% paraformaldehyde (PFA). Cells in serum-free medium were starved and then stimulated with the respective supplemented serum-free media, and fixed on day 1 with 4% PFA. Samples were permeabilized (0.1% Triton X-100 (Sigma-Aldrich, Buchs, Switzerland) in 1x PBS) and blocked with 3% bovine serum albumin (BSA) (Sigma-Aldrich, Buchs, Switzerland) for 1 h at room temperature. Afterward, rabbit anti ki-67 antibody (ab15580, Abcam, Cambridge, MA, 1:500 dilution) in 3% BSA was incubated with the samples at 4 °C, overnight. The next day, samples were washed three times with 1x PBS and incubated with secondary goat anti-rabbit Alexa555 antibody (A21249, Invitrogen, Carlsbad, CA, USA, 1:500 dilution) in 3% BSA in 1x PBS for 1 h at room temperature. Samples were washed and then incubated with Phalloidin (A12379, Invitrogen, Carlsbad, CA, USA, 1:500 dilution) for 1 h at room temperature, followed by a sample wash. In the end, samples were incubated with DAPI (5 μg/mL) for 10 min at room temperature, washed and imaged with confocal microscopy (SP5, Leica Microsystems, Wetzlar, Germany) using 20x/0.7 N.A. objective. Ten images from each sample (*n* = 3) were acquired.

### 4.4. RNA Isolation and Quantitative Real-Time PCR Analysis

In order to determine the effect of AA and PDGF-BB supplementation on the gene expression of tenocytes in vitro over time, rabbit tenocytes were isolated and cultured as described in the sub-section *Isolation of rabbit Achilles tenocytes* and seeded into 12-well plates (TPP, Trasadingen, Switzerland, growth area per well: 3.60 cm^2^) with a density of 4 × 10^4^ cells/ well. Cells were allowed to attach overnight before the medium was exchanged to medium with PDGF-BB (25 ng/mL) or AA (50 μg/mL) or without any supplementation (Ham’s F12, 10% FBS, 50 µg/mL gentamicin). Desired PDGF-BB and AA concentrations were added freshly to the medium every time during medium exchange, for the whole duration of the experiment. The medium was changed every second day, and cells were stimulated with PDGF-BB or AA for a total of 14 days. Samples were collected on day 3, 7 and 14. Rabbit tenocytes from four different animals were used (*n* = 4) for the experiments with PDGF-BB supplementation from three different animals (*n* = 3) for the experiments with AA supplementation. In all the experiments, triplicates of cell culture samples for each animal were run. At the respective time point, total RNA was isolated using the RNeasy Plus Mini Kit (Qiagen, Hilden, Germany) with RNase-free DNase treatment (Qiagen, Hilden, Germany), following the manufacturer’s protocol. For reverse transcription (RT), 500 ng of total RNA was reverse transcribed into cDNA in a reaction volume of 20 µL using the iScript™ Advanced cDNA Synthesis Kit (Bio-Rad, Cressier, Switzerland). Real-Time PCR reactions were performed on the resulting cDNA samples (5 ng cDNA per reaction), using the CFX Connect™ Real-Time PCR Detection System (Bio-Rad, Cressier, Switzerland) and SsoAdvanced™ SYBR^®^ Green Supermix (Bio-Rad, Cressier, Switzerland). The PCR reactions were incubated at 95 °C for 3 min, followed by 39 cycles of 95 °C for 10 s and 62 °C for 30 s. All samples were run in technical duplicates. The primer sequences used are outlined in Table S1. All primers were synthesized by Microsynth, Balgach, Switzerland. Relative expression analysis was performed using the comparative 2^−∆CT^ method with 18S rRNA as a reference gene, which was stable over the two conditions analyzed. Results are presented as fold change normalized to control, i.e., compared to samples cultivated without PDGF-BB or AA. Data is shown in box-plots, where the whiskers represent the standard deviation of the data.

### 4.5. Extracellular Matrix Production under Different Conditions

In order to test the effect of AA, PDGF-BB, or TGF-β1 supplementation on collagen I and fibronectin production in vitro, tenocytes were seeded into 8-well μ slides, ibiTreat (growth area per well: 1.0 cm^2^) at 7 × 10^4^ cells/mL (300 μL per well) in a cell culture medium. Cells were allowed to attach overnight, and the following day the cell culture medium was exchanged with cell culture medium supplemented with 50 ng/mL PDGF-BB or 10 ng/mL TGF-β (PeproTech, London, UK) or 50 μg/mL AA. Additionally, the cell culture medium in all conditions was supplemented with 30 μg/mL fibronectin (unlabeled) isolated from human plasma [59]. A culture medium supplemented only with fibronectin was used as a control condition. The culture medium was exchanged every 2 days. Desired PDGF-BB and AA concentrations were added freshly to the medium every time during medium exchange, for the whole duration of the experiment.

For immunofluorescence staining, separate samples were prepared and stained for collagen I and fibronectin deposition assessment. For collagen I staining, at the respective time points (day 3, 7 and 14), samples were washed with 1x PBS, and then culture medium with mouse anti-collagen I antibody (ab90395, Abcam, Cambridge, MA, USA, 1:200 dilution) was added to unfixed samples and incubated for 1 h at 37 °C. Afterward, samples were washed three times with 1x PBS (pH 7.4) and fixed for 10 min with 4% PFA and washed again. Samples were blocked with 3% BSA in 1x PBS for 1 h. Secondary donkey anti-mouse Alexa-488 antibody (A-21202, Invitrogen, Carlsbad, CA, USA, 1:500 dilution) in 3% BSA in 1x PBS was added afterward and incubated for 1 h at room temperature. Nuclei were stained with 5 μg/mL DAPI for 10 min and washed afterward. For fibronectin staining, the samples were washed with 1x PBS, fixed for 10 min with 4% PFA, and washed again. Afterward, they were blocked with 3% BSA in 1x PBS for 1 h and subsequently incubated with mouse anti-fibronectin antibody (F0791, Sigma-Aldrich, Buchs, Switzerland, 1:200 dilution) overnight at 4 °C. The next day, samples were washed and incubated with secondary donkey anti-mouse Alexa-488 (A-21202, Invitrogen, Carlsbad, CA, USA 1:500 dilution) antibody for 1 h at room temperature. Nuclei were stained with 5 μg/mL DAPI for 10 min and washed afterwards. Samples were imaged with confocal microscopy (SP5, Leica Microsystems, Wetzlar, Germany) with 20x/ 0.7 N.A. objective, and z-stacks were acquired. Four images per sample (*n* = 3) for each condition were acquired. Confocal images are presented as maximum projections of the z-stacks.

### 4.6. Tenocyte Culture on Electrospun Scaffolds with Incorporated AA or PDGF-BB

Emulsion electrospun scaffolds made from DegraPol^®^ (DP) (Ab medica, Milan, Italy) with incorporated PDGF-BB (diluted in 0.1% BSA in distilled water at a concentration of 40 μg/mL) were produced as reported previously [47]. For the production of emulsion electrospun scaffolds with incorporated AA, AA was dissolved in water at a concentration of 50 mg/mL and 200 μL of this stock solution were added drop wise to 5 g of 12 wt% DP polymer solution (dissolved in mixture of chloroform/HFP (80:20) (Sigma-Aldrich, Buchs, Switzerland), under stirring for 2 min at 500 rpm. Afterwards the mixture was sonicated with a probe ultrasonicator for 2 min at 50% amplitude. Immediately afterward, the emulsion was used for electrospinning as reported previously [47].

After electrospinning, the scaffolds were dried in vacuum overnight. Before cell seeding, scaffolds were UV sterilized for 30 min. Afterward, scaffolds were shortly wetted in 50% ethanol, cut in small squares that could fit in one well of 8-well μ slides, ibiTreat (growth area per well: 1.0 cm^2^), rinsed three times in water, put into the 8-well μ slides, and washed one time with culture medium. Culture medium was removed from each well, and tenocytes with a density of 4 × 10^4^ per well were seeded on top of the scaffolds containing AA, PDGF-BB, or no biomolecule (pure scaffolds) (*n* = 3). The culture medium, containing 30 μg/mL fibronectin, was changed every 2 days. At each respective time point (day 3, 7 or 14), the culture medium was aspirated, samples were washed one time with 0.1 M PIPES (pH 7.4) buffer and samples were fixed in 4% glutaraldehyde (Sigma-Aldrich, Buchs, Switzerland) at 4 °C, overnight. The next day, samples were washed in 0.1 M PIPES buffer and dehydrated by placing them in a series of ascending concentration of ethanol (30%, 50%, 70%, 80%, 95% (5 min each step), and 100% (10 min, twice)). Afterwards, the samples were chemically dried in HMDS/ethanol mix (3:1, 1:1, 1:3 and pure HMDS, each for 15 min). After allowing the HMDS to evaporate completely overnight, the samples were mounted on SEM stubs, sputter coated (SCD500, Bal-tec, Balzers, Lichtenstein) with platinum in order to obtain 10 nm coating and then examined by scanning electron microscope (Zeiss SUPRA 50 VP, Zeiss, Cambridge, UK) at an accelerating voltage of 5 kV.

For assessing collagen I and fibronectin deposition onto the electrospun scaffolds, cells were seeded in the same way as for the scaffolds for SEM evaluation. Culture medium containing 30 μg/mL fibronectin was replenished every 2 days and the samples were fixed with 4% PFA on day 3, 7 and 14. Staining of collagen I and fibronectin was performed in the same way as described in sub-Section 4.5 *Extracellular matrix production under different conditions.* Eventually, the scaffolds were mounted on glass slides using DAKO Fluorescent mounting medium and covered with glass coverslips. Z-stack images were taken with confocal microscope (SP5, Leica Microsystems, Wetzlar, Germany) with 40x objective. Confocal images are presented as maximum projections of the z-stacks.

### 4.7. Statistical Analysis

Data were analyzed with Origin (OriginPro 2017, OriginLab, Northampton, MA, USA). Values were expressed as means ± standard deviation. For the data from the proliferation experiments, one-way analysis of variance (one-way ANOVA) was performed to test the significance of differences between different groups using a comparison post hoc test for significance. T-test was performed to analyze the differences between the conditions in the gene expression experiments between groups treated with PDGF-BB or AA, respectively, and non-treated controls. Statistical significance was accepted for *p* < 0.05 marked by an asterisk (*).

## Supplementary Materials

Supplementary materials can be found at https://www.mdpi.com/1422-0067/21/2/458/s1.

## Abbreviations

AA	Ascorbic acid
bFGF	Basic fibroblast growth factor
IGF	Insulin-like growth factor
MSCs	Mesenchymal stem cells
MMPs	Matrix metalloproteases
PDGF-BB	Platelet-derived growth factor-BB
TGF-β1	Transforming growth factor-beta 1
TIMPs	Tissue inhibitors of metalloproteases
VEGF	Vascular endothelial growth factor
DP	DegraPol^®^ polymer
