# Supplementary material for: Supporting Cell-Based Tendon Therapy: Effect of PDGF-BB and Ascorbic Acid on Rabbit Achilles Tenocytes In Vitro
Source: Int J Mol Sci. 2020 Jan 10;21(2):458. doi: 10.3390/ijms21020458 (PMC7014238; doi:10.3390/ijms21020458)
Supplement: Supplementary file 1 [file ijms-21-00458-s001.pdf]

Supporting Information

Table S1. Gene abbreviations, NCBI reference sequences of mRNAs (used as template for primer design), amplicon size and primer sequences used for Real-Time PCR Analysis.

Gene	mRNA ID (NCBI Reference Sequence)	Amplicon Size (bp)	Forward Primer Sequence (3′→ 5′)	Reverse Primer Sequence (5′→ 3′)
18S rRNA	NR_033238.1	176	GGAACTGAGGCCATGATTAAG	CGGAACTACGACGGTATCTG
COL1A1	XM_008271783.1	271	CTGGTGAATCTGGACGTGAG	TGTCTCACCCTTGTCACCAC
COL1A2	NM_001195668.1	170	CCAATCACGCCTCTCAGAAC	GCAGCCATCGACAAGAACAG
COL3A1	XM_002712333.2	259	GCATTGCTTACATGGATCAGG	CCAACGTCCGCACCAAATTC
FN	AF135404.1	270	GCAACCCACAGTGGAATACG	AGTCCTGACACAACAACAGAC
αSMA	NM_001101682.2	224	CGTGACTACTGCTGAACGTG	GGATGCCAGCAGATTCCATC
TNC	FJ480400.1	219	GTCACTCATCACAGCTCTGG	CTGAGTGTGTATTCCGTGGC
TNMD	NM_001109818.1	239	GCAGTTTCCGAGTTACAAGAC	CGACGGCAGTAAATACAACAG
DCN	NM_001082330.1	255	GTGGACAATGGTTCTCTGGC	AAGGTGGATGGCTGGATCTC
ACAN	L38480.1	113	GCTACGGAGACAAGGATGAG	GTAAAAGACCTCACCCTCCAT
BGN	NM_001195691.1	199	GGCCTGAAGCTCAACTACCT	GGCTCCCGTTCTCAATCATC
MKI67	XM_008251084.2	283	CACATCCAGCAGTGAAACGG	GTGTTAGCAGTACCTGAAGTC
MMP2	D63579.1	220	GAAGATCGACGCTGTGTACG	GTATCTCCAGAACTTGTCTCC
MMP9	D26514.1	160	GATACAGCCTGTTCCTCGTG	GGACCATATAGATGCTGGATG
TIMP1	NM_001082232.2	236	CTACCTTGTACCAGCGTTATG	GAAGCTCAGACTGTTCCAGG
TIMP2	XM_008252510.2	224	CTGTCTGGAGGAAGCACTTG	CTGCTCAGCGTGGATACAAG

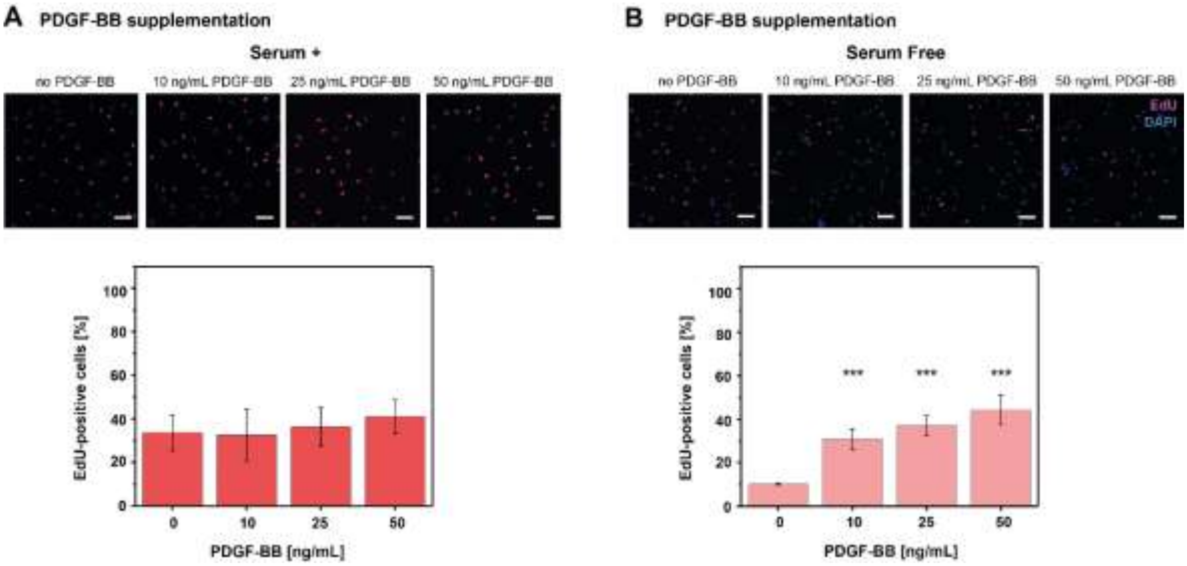

Figure S1. Effect of PDGF-BB supplementation on tenocyte proliferation. Representative CLSM images from the EdU proliferation assay and EdU-positive cells [%] for each PDGF-BB concentration (ng/mL) tested in A) serum+ conditions and B) serum-free conditions. Data was adapted from [47]. The total EdU-positive cells [%] was calculated as EdU positive cells (pink) in relation to the total cell number (DAPI stained, blue). Scale bars: 100 µm. (Data shown as mean ± standard deviation, ** $p < 0.01$, ***$p < 0.001$, obtained by one-way ANOVA).

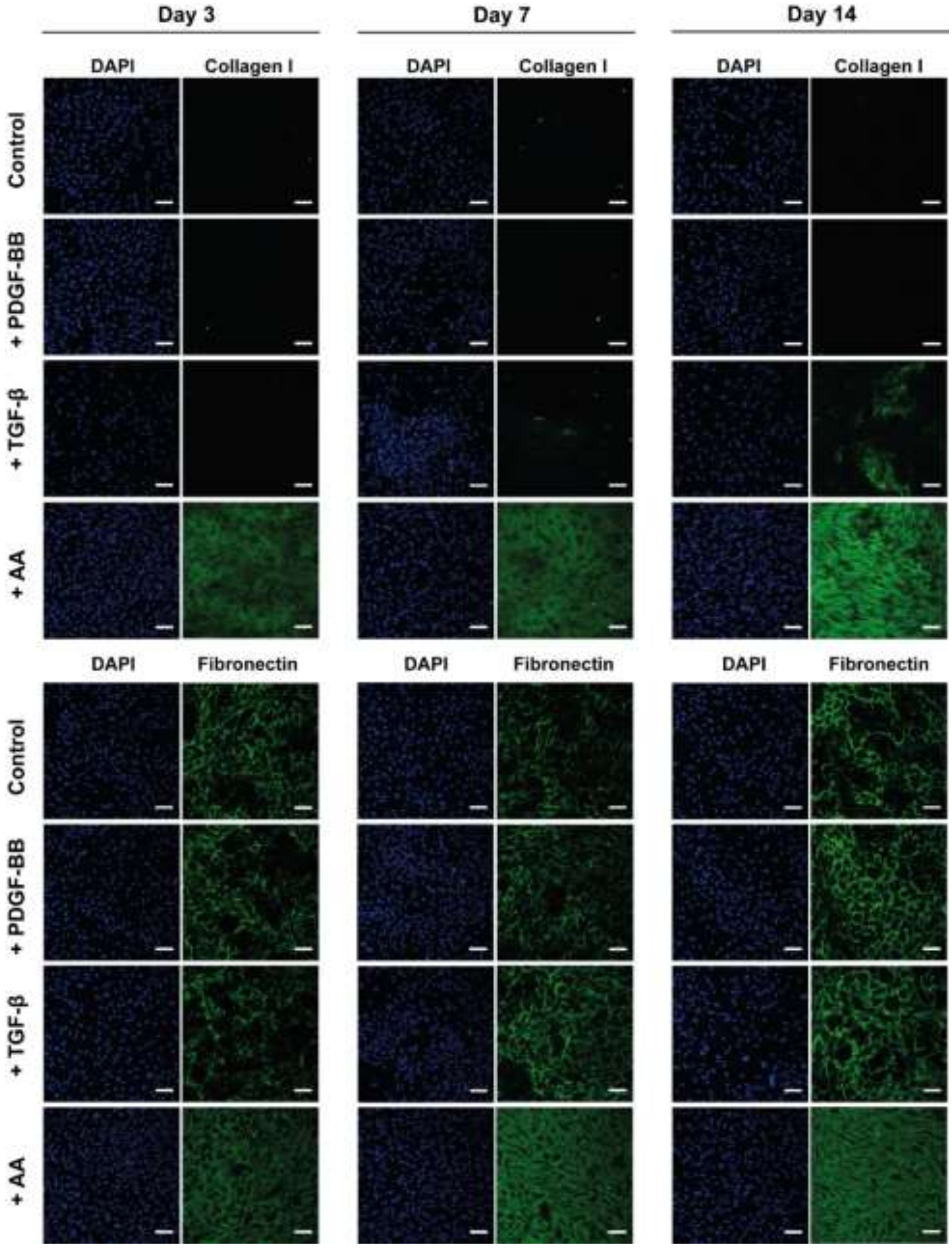

Figure S2. Collagen I and fibronectin staining (green) and cell nuclei DAPI staining (blue) of rabbit tenocytes (Animal 2) cultured in vitro in 2D, treated with PDGF-BB, TGF-β, AA or untreated (control) at day 3, 7 and 14. Scale bars: 100 µm. .

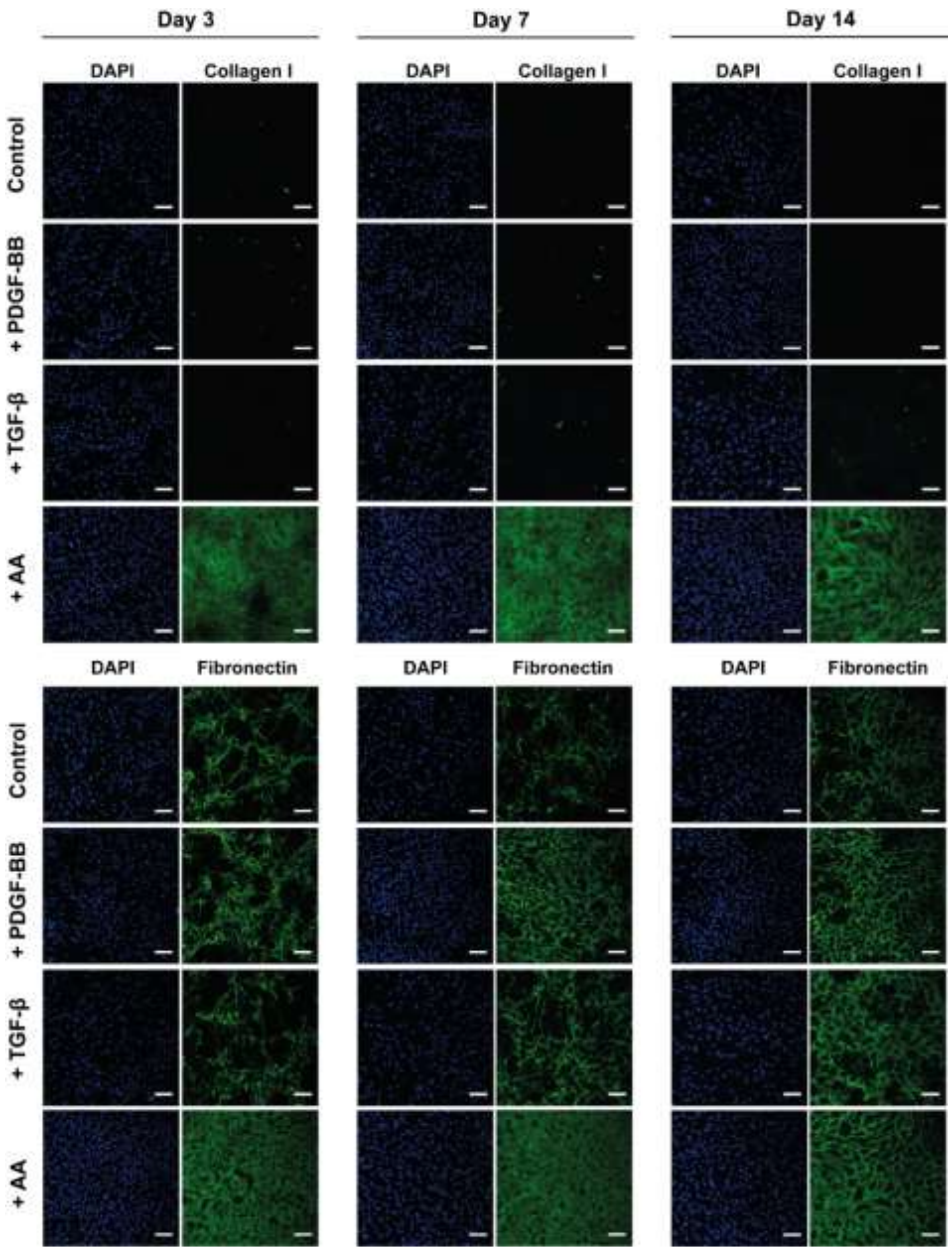

Figure S3. Collagen I and fibronectin staining (green) and cell nuclei DAPI staining (blue) of rabbit tenocytes (Animal 3) cultured in vitro in 2D, treated with PDGF-BB, TGF-β, AA or untreated control at day 3, 7 and 14. Scale bars: 100 μm.

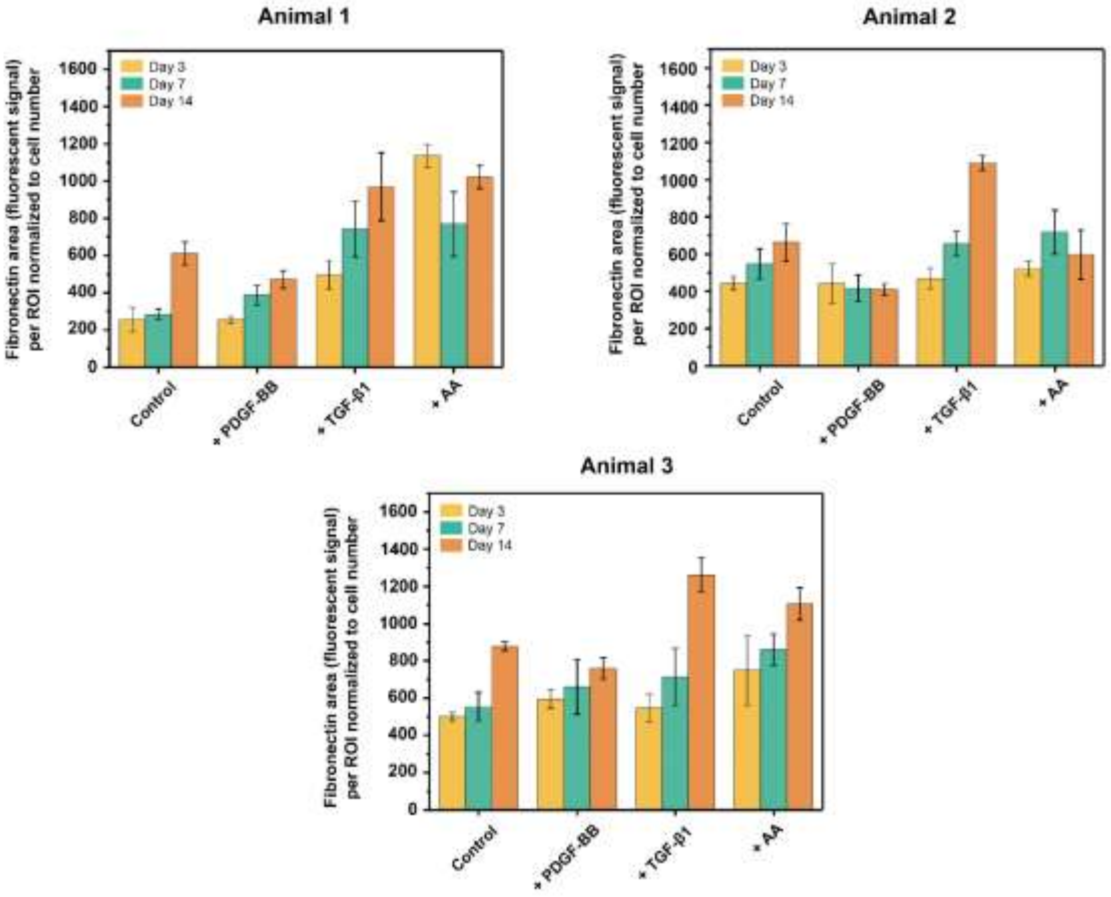

Figure S4. Quantification of fibronectin deposition by tenocytes in the different treatment conditions. The signal of fibronectin staining was assessed from images of maximum projections of the z-stacks obtained with confocal microscopy. Briefly, thresholded images were segmented and the total area of the signal in each image was assessed. The area measured was normalized to the cell number relative values for the ratio obtained are shown on the y-axis) in each image and thus the fibronectin deposition was quantified independent of cell density, as the treatments used do increase cell proliferation.
